# The Evolutionary Genetics and Emergence of Avian Influenza Viruses in Wild Birds

**DOI:** 10.1371/journal.ppat.1000076

**Published:** 2008-05-30

**Authors:** Vivien G. Dugan, Rubing Chen, David J. Spiro, Naomi Sengamalay, Jennifer Zaborsky, Elodie Ghedin, Jacqueline Nolting, David E. Swayne, Jonathan A. Runstadler, George M. Happ, Dennis A. Senne, Ruixue Wang, Richard D. Slemons, Edward C. Holmes, Jeffery K. Taubenberger

**Affiliations:** 1 Laboratory of Infectious Diseases, National Institute of Allergy and Infectious Diseases, National Institutes of Health, Bethesda, Maryland, United States of America; 2 Center for Infectious Disease Dynamics, Department of Biology, The Pennsylvania State University, University Park, Pennsylvania, United States of America; 3 The J. Craig Venter Institute, Rockville, Maryland, United States of America; 4 Department of Medicine, School of Medicine, University of Pittsburgh, Pittsburgh, Pennsylvania, United States of America; 5 Department of Veterinary Preventive Medicine, The Ohio State University, Columbus, Ohio, United States of America; 6 Southeast Poultry Research Laboratory, Agricultural Research Service, US Department of Agriculture, Athens, Georgia, United States of America; 7 Institute of Arctic Biology, University of Alaska Fairbanks, Fairbanks, Alaska, United States of America; 8 National Veterinary Services Laboratories, Animal and Plant Health Inspection Service, US Department of Agriculture, Ames, Iowa, United States of America; 9 Fogarty International Center, National Institutes of Health, Bethesda, Maryland, United States of America; University of Maryland, United States of America

## Abstract

We surveyed the genetic diversity among avian influenza virus (AIV) in wild birds, comprising 167 complete viral genomes from 14 bird species sampled in four locations across the United States. These isolates represented 29 type A influenza virus hemagglutinin (HA) and neuraminidase (NA) subtype combinations, with up to 26% of isolates showing evidence of mixed subtype infection. Through a phylogenetic analysis of the largest data set of AIV genomes compiled to date, we were able to document a remarkably high rate of genome reassortment, with no clear pattern of gene segment association and occasional inter-hemisphere gene segment migration and reassortment. From this, we propose that AIV in wild birds forms transient “genome constellations,” continually reshuffled by reassortment, in contrast to the spread of a limited number of stable genome constellations that characterizes the evolution of mammalian-adapted influenza A viruses.

## Author Summary

Influenza A viruses are an extremely divergent group of RNA viruses that infect in a variety of warm-blooded animals, including birds, horses, pigs, and humans. Since they contain a segmented RNA genome, mixed infection can lead to genetic reassortment. It is thought that the natural reservoir of influenza A viruses is the wild bird population. Influenza A viruses can switch hosts and cause novel outbreaks in new species. Influenza viruses containing genes derived from bird influenza viruses caused the last three influenza pandemics in humans. In this study, we surveyed the genetic diversity among influenza A viruses in wild birds. Through a phylogenetic analysis of the largest data set of wild bird influenza genomes compiled to date, we were able to document a remarkably high rate of genome reassortment, with no clear pattern of gene segment association and occasional inter-hemisphere gene segment migration and reassortment. From this, we propose that influenza viruses in wild birds forms transient “genome constellations,” continually reshuffled by reassortment, in contrast to the spread of a limited number of stable genome constellations that characterizes the evolution of mammalian-adapted influenza A viruses.

## Introduction

Low pathogenic (LP), antigenically diverse influenza A viruses are widely distributed in wild avian species around the world. They are maintained by asymptomatic infections, most frequently documented in aquatic birds of the orders Anseriformes and Charadriformes. As such, wild birds represent major natural reservoirs for influenza A viruses [1]–[11] and at least 105 species of the more than 9000 species of wild birds have been identified as harboring influenza A viruses [8],[12],[13]. These influenza A viruses, commonly referred to as avian influenza viruses (AIV), possess antigenically and genetically diverse hemagglutinin (HA) [14] and neuraminidase (NA) subtypes, which includes all known influenza A virus HA (H1–H16) and NA (N1–N9) subtypes. At least 103 of the possible 144 type A influenza A virus HA-NA combinations have been found in wild birds [8],[15].

AIV maintained in wild birds have been associated with stable host switch events to novel hosts including domestic gallinaceous poultry, horses, swine, and humans leading to the emergence of influenza A lineages transmissible in the new host. Adaptation to domestic poultry species is the most frequent [16]–[26]. Sporadically, strains of poultry-adapted H5 or H7 AIV evolve into highly pathogenic (HP) AIV usually through acquisition of an insertional mutation resulting in a polybasic amino acid cleavage site within the HA [15],[25]. The current panzootic of Asian-lineage HP H5N1 AIV appears to be unique in the era of modern influenza virology, resulting in the deaths of millions of poultry in 64 countries on three continents either from infection or culling. There are also significant zoonotic implications of this panzootic, with 379 documented cases in humans, resulting in 239 deaths in 14 countries since 2003 (as of April 2008 [27]). The Asian lineages of HP H5N1 AIV have also caused symptomatic, even lethal, infections of wild birds in Asia and Europe, suggesting that migratory wild birds could be involved in the spread of this avian panzootic [28]–[31]. Concern is heightened since wild birds are also likely to be the reservoir of influenza A viruses that switch hosts and stably adapt to mammals including horses, swine, and humans [3]. The last three human influenza pandemic viruses all contained two or more novel genes that were very similar to those found in wild birds [16],[20],[32],[33].

Despite the recent expansion of AIV surveillance [7],[8],[10],[34],[35] and genomic data [5], [36]–[38], fundamental questions remain concerning the ecology and evolution of these viruses. Prominent among these are: (i) the structure of genetic diversity of AIV in wild birds, including the role played by inter-hemispheric migration, (ii) the frequency and pattern of segment reassortment, and (iii) the evolutionary processes that determine the antigenic structure of AIV, maintained as discrete HA and NA subtypes. Herein, we address these questions using the largest data set of complete AIV genomes compiled to date.

## Results/Discussion

### Global Genome Diversity of AIV

The complete genomes of 167 influenza A viruses isolated from 14 species of wild Anseriformes in 4 locations in the U.S. (Alaska, Maryland, Missouri, and Ohio) were sequenced; viral isolates consisted of 29 HA and NA combinations, including 11 HA subtypes (H1–H8, H10–H12) and all 9 neuraminidase subtypes (N1–N9). These sequences were collected as part of an ongoing AIV surveillance project at The Ohio State University and collaborators in other states (1986–2005) using previously described protocols [39], and more than double the number of complete U.S.-origin avian influenza virus genomes available in GenBank. In total, 1340 viral gene segment sequences (2,226,085 nucleotides) were determined (Table S1) and are listed on the Influenza Virus Resource website (http://www.ncbi.nlm.nih.gov/genomes/FLU/Database/shipment.cgi).

Cloacal samples from wild birds frequently show evidence of mixed infections with influenza viruses of different subtypes by serologic analysis [39]–[41]. Therefore, the isolates chosen for sequence analysis were subjected to sequential limiting dilutions (SLD) [39]. The amplification and sequencing pipeline employed a ‘universal’ molecular subtyping strategy in which every sample was amplified with sets of overlapping primers representing all HA and NA subtypes. In this manner, samples without clear prior subtype information, and/or mixed samples, could be accurately analyzed. Despite performing SLD, 4 samples were shown by sequence analysis to represent a mixed infection (yielding sequence with more than one HA and/or NA subtype. In addition 40 samples had mismatches between the initial antigenic subtyping results (determined on first- or second-egg-passage isolates prior to SLD) and the subtype determined by sequence analysis of cDNA (following one SLD of low-egg-passage isolates) which suggests the possibility of minor populations of antigenically distinct viruses in the low-passage isolate that outgrew the dominant antigenic population in a foreign host system during the SLD or that mixed infections in first egg passage stock caused difficulty in initial subtyping and a dominant strain emerged during SLD (see table of viral isolates at http://www.ncbi.nlm.nih.gov/genomes/FLU/Database/shipment.cgi to examine the discordant results observed). Thus, up to 44 of 167 (26%) of isolates potentially represent mixed infections in the initial cloacal sample. Given the SLD procedure, the true rate of mixed infection, as defined by the presence of >1 HA and/or NA subtype, was likely to be even higher, although mis-serotyping cannot also be ruled out. Sequencing viral genomes directly from primary cloacal material would be the only way to assess the mixed infection frequency, in a manner unbiased by culture, but no such studies have yet been attempted to our knowledge.

**Figure 1:**
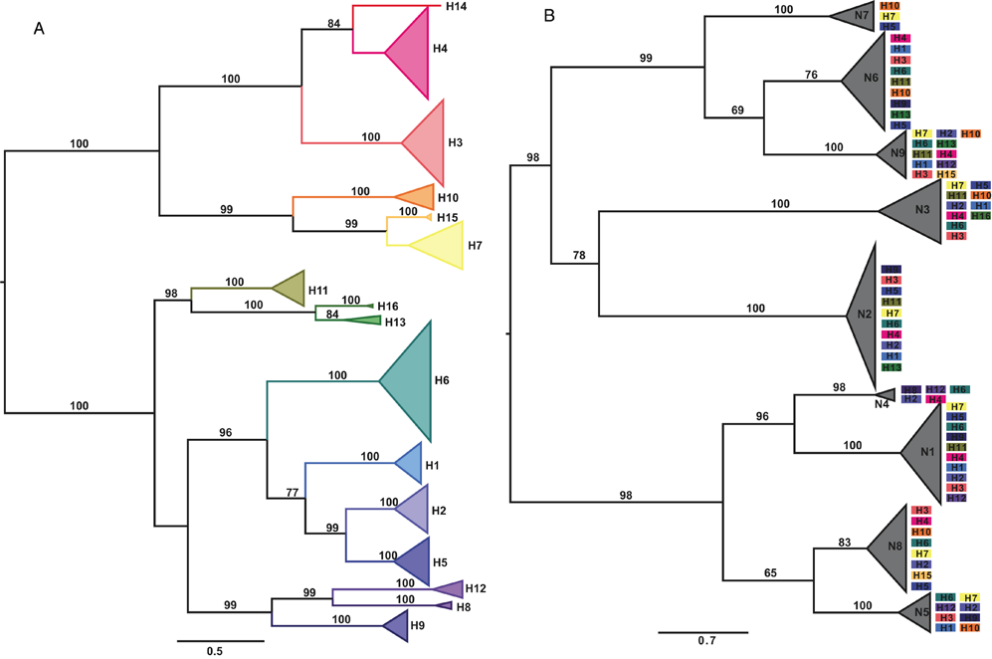
Maximum likelihood trees of HA and NA genes. (a) Maximum likelihood tree of the HA gene segment of 452 isolates of avian influenza A virus, including representatives of all 16 subtypes. For clarity, all branches within individual subtypes have been collapsed and color-coded to signify individual subtypes. Bootstrap values above 70% are shown next to relevant branches. Branch lengths are scaled according to the number of nucleotide substitutions per site. See Figure S1 for an expanded form of this tree. (b) Maximum likelihood tree of the NA gene segment of 473 isolates of avian influenza A virus, including representatives of all 9 subtypes. The mix of HA subtypes (color-coded according to Figure 1a) observed within each NA type is shown, highlighting the frequency of reassortment. For clarity, all branches within individual subtypes have been collapsed. Bootstrap values above 65% are shown next to the relevant branches. Branch lengths are scaled according to the number of nucleotide substitutions per site. See Figure S2 for an expanded form of this tree, in which individual viral isolates are marked.

For a more comprehensive analysis of AIV diversity, the AIV genomes from this study were compared to other AIV genomes available on GenBank [38]. In total, 452 HA sequences and 473 NA sequences, representative of the global diversity of AIV, were used in phylogenetic analyses. For the internal protein genes (PB2, PB1, PA, NP, M, NS), a subset of 407 complete globally-sampled AIV genomes was used to assess the degree of linkage among gene segments. Phylogenetic trees for the HA alignment (Figures 1a and S1) and NA alignment (Figure 1b and S2) are shown here. Phylogenetic trees for the six other gene segments are presented in Figures S3, S4, S5, S6, S7 and S8.

The topology of the HA phylogeny reflects the antigenically defined subtypes, with some higher-order clustering among them (e.g., H1, H2, H5 and H6; H7, H10 and H15; Figures 1a and S1), as seen previously in smaller studies [14],[42]. Although most subtypes are found in numerous avian species and occupy wide global distributions, this phylogenetic structure indicates that HA subtypes did not originate in a single radiation. More striking was the high level of genetic diversity between different subtypes; the average amino acid identity of 120 inter-subtype comparisons of full-length HA was 45.5%. As expected, inter-subtype comparisons of the HA1 domain exhibited more diversity, with an average inter-subtype identity of 38.5%. In contrast, intra-subtype identity is high (averaging >92%), even when comparing sequences from different hemispheres. Hence, the genetic structure of the AIV HA is characterized by highly divergent subtypes that harbor relatively little internal genetic diversity. However, 4 subtype comparisons show considerably less divergence (76–79% identity); H4 vs. H14, H7 vs. H15, H13 vs. H16, and H2 vs. H5, indicating that they separated more recently (Figure 1; see below).

A similar phylogenetic structure was seen in the NA (Figure 1b and S2), again with evidence for higher-order clustering (e.g., N6 and N9; N1 and N4). In contrast to the HA, however, levels of genetic divergence among the NA types are more uniform, with the 9 subtypes exhibiting an average inter-subtype identity of 43.6% (with an average intra-subtype identity of >89%) and no clear outliers. Hence, no new (detected) NA types have been created in the recent evolutionary past. This correlates with the more uniform distribution of NA than HA subtypes in wild bird AIV isolates [43].

The topology of the NS segment phylogeny was also of note, being characterized by the deep divergence among the A and B alleles as described [44] (Figure S8). Almost every HA and NA subtype of AIV contain both the A and B NS alleles, without evidence of ‘intermediate’ lineages expected under random genetic drift, strongly suggesting that the two alleles are subject to some form of balancing selection. The NS1 protein has pleiotropic functions during infection in mammalian cells, and plays an important role in down-regulating the type I interferon response [45]. Supporting these results are the elevated rates of nonsynonymous to synonymous substitution per site (ratio d_N_/d_S_) observed for the NS1 gene in both avian and human influenza viruses [46] suggesting that the NS1 protein has experienced adaptive evolution in both host types. Whether this selection relates to the role the NS1 protein plays in its interaction in the type I interferon pathway is currently unclear.

Far less genetic diversity is observed in the 5 remaining AIV gene segments (PB2, PB1, PA, NP, and M - Figures S3, S4, S5, S6 and S7). Indeed, the extent of diversity in these genes is less than that *within* a single HA or NA subtype, with average pairwise identities ranging from 95–99%. Our phylogenetic analysis also revealed a clear separation of AIV sequences sampled from the Eastern and Western Hemispheres, as previously noted (3,19), indicating that there is relatively little gene flow between overlapping Eastern and Western Hemisphere flyways. However, despite this strong biogeographic split, mixing of hemispheric AIV gene pools clearly occurs at a low level (see below).

### Abundant reassortment in AIV

**Figure 2:**
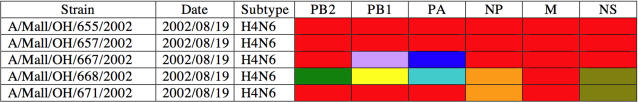
The genome constellations (internal gene segments only) of 5 H4N6 viruses collected from mallards at the same location in Ohio, USA on the same day. The different colors reflect segments whose sequences fall into different major clades – defined by strong bootstrap support (>80%) – in each internal gene segment tree. For example, all 6 internal gene segments from isolates A/Mall/OH/655/2002 and A/Mall/OH/657/2002 have the same, shared phylogenetic position (shaded red), but exhibit a significantly different phylogenetic pattern, indicative of reassortment, with A/Mall/OH/667/2002 in the PB1 and PA gene segments (individual trees presented in Figures S4 and S5). Similarly, isolate A/Mall/OH/668/2002 shows phylogenetic evidence of reassortment in 5 of 6 internal gene segments compared to A/Mall/OH/655/2002.

To assess the frequency and pattern of reassortment in AIV, we compared the extent of topological similarity (congruence) among phylogenetic trees of each internal segment. This analysis revealed a remarkably frequent occurrence of reassortment, supporting previous studies on smaller data sets [37],[47]. For example, 5 H4N6 AIV isolates were recovered from mallards sampled at the same location in Ohio on the same morning and in the same trap (Figure 2). For the internal genes, these viruses contained 4 different genome ‘constellations’, with only 1 pair of viruses sharing the same constellation. In the data set as a whole, the large number of different subtype combinations recovered highlights the frequency of reassortment (Figures 1b and S2), and provides little evidence for the elevated fitness of specific HA/NA combinations in AIV isolates from wild birds. That the majority of HA/NA combinations have been recovered [8],[15] also strongly supports the high frequency of reassortment involving these surface protein genes.

**Figure 3:**
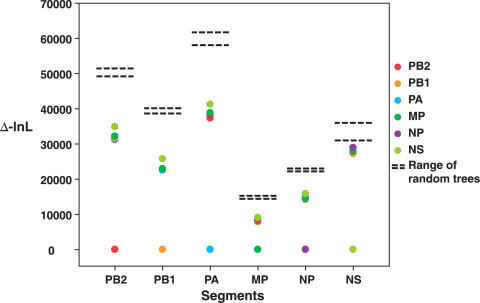
Maximum likelihood analysis of congruence among the internal gene segments of 407 isolates of avian influenza virus. Each column represents the difference in log likelihood (Δ-lnL) between the ML trees of each gene (shown by colored dots). In every case, the ML tree estimated for the reference gene has the highest likelihood, while lower likelihoods (greater Δ-lnL values) are observed when the ML trees for the other genes are fitted to the sequence data from the reference gene and branch lengths re-optimized. To assess the extent of similarity in topology among genes, 500 random trees were created for each data set and their likelihoods assessed for each gene in turn using the same procedure (indicated by horizontal bars). In every case, and most notably for NS, the trees inferred for each gene have likelihoods closer to the random set than to the ML tree for the reference gene, indicative of extensive incongruence.

Thus, while there is strong evidence of frequent reassortment between HA and NA, we also sought to assess the extent of reassortment among the less commonly studied internal gene segments. A maximum likelihood test of phylogenetic congruence [48] revealed that although the topologies of the internal segment trees are more similar to each other than expected by chance, so that the segments are not in complete linkage equilibrium (in which case they would be no more similar in topology than two random trees), the difference among them is extensive, indicative of extremely frequent reassortment and with little clear linkage among specific segments (Figure 3). Of the 6 internal segments, NS exhibited the least linkage to other genes, falling closest to the random distribution (i.e. possessed the greatest phylogenetic incongruence). This is compatible with the deep A and B allelic polymorphism in this segment. In contrast, the M segment showed the greatest phylogenetic similarly, albeit slight, to the other segments. Overall, however, the relationships between segments are better described by their dissimilarity than their congruence.

Occasional AIV isolates demonstrated hemispheric mixing with reassortment. As reported previously, the majority of such mixing occurs in shorebirds and gulls [36] (with the exception of Eurasian lineage H6 HA genes distributed widely in North American Anseriformes [5] as also revealed in this study). Interestingly, no completely Eurasian-lineage AIV genome has been reported in North America, or *vice versa* [9],[49]. This suggests that birds initially carrying AIV between the hemispheric flyways have not been identified in surveillance efforts. Most mixed isolates possess only one gene segment derived from the other hemisphere, indicating that there is little or no survival advantage for such hemispheric crossovers in the new gene pool. Since Asian lineage HP H5N1 AIV have been isolated from wild birds in Eurasia [50], concern has been raised over the importation of the virus into North America via migratory birds. Our analyses suggest that enhanced surveillance in gulls and other shorebirds may be warranted, and that with frequent reassortment (see below), entire Asian HP H5N1 AIV isolate genome constellations may not be detected in these surveys.

Overall, 25 of 407 (6%) AIV genomes show evidence of hemispheric mixing, with the phylogenies suggesting a general pattern of viral gene flow from Eurasia to North America: 5 North American isolates possessed two Eurasian-lineage internal gene segments, and 20 carried a single segment. North American isolates possessing a Eurasian-lineage M segment were the most common, seen in 18 isolates (Figure S7), followed by 8 with a Eurasian PB2 segment (Figure S3), four with a Eurasian PB1 segment (Figure S4), and 1 with a Eurasian PA segment (Figure S5). The 18 Eurasian M segments and the 8 Eurasian PB2 segments each form monophyletic groups, suggesting single introductions to North America. In each case, sequences from domestic ducks in China and turkeys in Europe were the closest relatives. It is therefore theoretically possible that some of these introductions may have been derived from imported poultry rather than migratory birds. In contrast, 3 of the 4 Eurasian PB1 and the single Eurasian PA segment in North American AIV contained genes whose closest relatives were in viruses found in red-necked stints from Australia. These small waders are widely migratory, with a range from Siberia to Australasia, and occasionally in Europe and North America. Interestingly, 23 of 25 such mixed genomes were observed in shorebirds along the U.S. Atlantic coast. Unfortunately, no complete AIV genomes are available from shorebirds on the U.S. Pacific coast for comparison.

### The Evolutionary Genetics of AIV

In theory, two evolutionary models can explain the global pattern of AIV diversity, analogous to the allopatric and sympatric models of speciation. Under the allopatric model, the HA and NA subtypes correspond to viral lineages that became geographically isolated, resulting in a gradual accumulation of amino acid changes among them. Because of physical separation through geographical divergence, there is no requirement for natural selection to reinforce the partition of HA and NA diversity into discrete subtypes by preferentially favoring mutations at antigenic sites. In contrast, under the sympatric model, the discrete HA and NA subtypes originate within the same spatial population, such that natural selection must have reinforced speciation; subtypes that were too antigenically similar would be selected against because of cross-protective immune responses. Therefore, mutations would accumulate first at key antigenic sites, allowing subtypes to quickly diversify in the absence of herd immunity.

**Figure 4:**
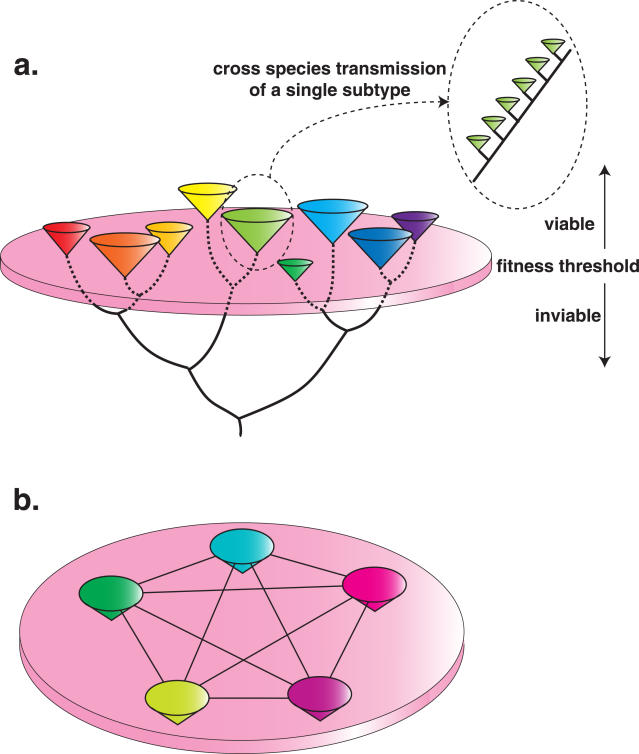
The fitness landscapes of avian influenza virus. (a) The fitness landscapes observed in HA, NA and NS, and represented here by NA. Each colored cone represents an individual subtype. These subtypes are connected by a bifurcating tree. The lack of ‘intermediate’ subtypes – those falling below the pink disc – reflects major valleys in fitness, such that any virus falling in this area will experience a major reduction in fitness, most likely due to an elevated cross-protective immune response. Occasionally, individual subtypes jump species barriers and spread in new hosts (such as humans), where they experience a continued selection pressure and hence accumulate amino acid substitutions in a progressive manner, as shown. (b) The fitness landscapes observed in the remaining internal protein segments of avian influenza virus – PB2, PB1, PA, NP and M (represented by different colors). In this case, there is little functional difference among the genetic variants of each segment, so that the fitness landscape is flat. This equivalence in fitness among genome constellations also means that reassortment is frequent among them (as reassortants suffer no fitness cost), represented by the horizontal lines connected each internal gene segment.

The AIV genomic data available here suggest a complex interplay of evolutionary processes. That discrete HA and NA subtypes, as well as the 2 divergent NS alleles, are maintained in the face of frequent reassortment strongly suggests that each represents a peak on a fitness landscape shaped by cross-immunity (Figure 4a). Under this hypothesis, ‘intermediate’ HA/NA/NS alleles would be selected against because they generate more widespread herd immunity, corresponding to fitness valleys. Indeed, it is the likely lack of immunological cross-protection at the subtype level that allows the frequent mixed infections described here (although mixed infections may also occur in young, immunologically naïve birds). Further, in most cases these divergent HA, NA and NS alleles circulate in the same bird species in the same geographical regions, compatible with their divergence under sympatry. In addition, 3 of the most closely related pairs of HA subtypes contain an HA that is rarely isolated or limited geographically or by host species restriction, implying that their dispersion is inhibited by existing immunity; H14 has only been isolated rarely in Southern Russia, H15 only in Australia, and H16 has only been described in gulls. The possible exception is H2–H5, where both subtypes have been isolated from a variety of bird species in a global distribution. Although these may represent more recent occurrences of allopatric speciation, antigenic cross-reactivity between the H2–H5, H7–H15, H4–H14 pairs was recently demonstrated [51], again compatible with the sympatric model. Further support for possible cross-immunity between these subtypes would require experimental challenge studies.

In contrast to the extensive genetic diversity seen in HA, NA and NS, the 5 remaining internal gene segments encode proteins that are highly conserved at the amino acid level, indicating that they are subject to widespread purifying selection. The fitness landscape for these genes is therefore not determined by cross-immunity, but by functional viability, with less selective pressure to fix advantageous mutations (Figure 4b). Further, given such strong conservation of amino acid sequence, large-scale reassortment is permitted as it will normally involve the exchange of functionally equivalent segments, with little impact on overall fitness. These data also suggest that the cross-immunity provided by these proteins is minimal.

Together, these global genomic data provide new insight into the different evolutionary dynamics exhibited by influenza A viruses in their natural wild bird hosts and in those viruses stably adapted to novel species (e.g., domestic gallinaceous poultry, horses, swine, and humans). Based on these analyses, we hypothesize that AIV in wild birds exists as a large pool of functionally equivalent, and so often inter-changeable, gene segments that form transient genome constellations, without the strong selective pressure to be maintained as linked genomes. Rather than favoring successive changes in single subtypes, geographic and ecologic partitioning within birds, particularly within the different flyways, coupled with complex patterns of herd immunity, has resulted in an intricate fitness landscape comprising multiple fitness peaks of HA, NA and NS alleles, interspersed by valleys of low fitness which prevent the generation of intermediate forms (Figure 4a).

In contrast, stable host switching involves the acquisition of a number of (as yet) poorly characterized mutations [24],[33],[52],[53] that serve to separate an individual, clonally derived influenza virus strain from the large wild bird AIV gene pool. Because adaptation to a new host likely limits the ability of these viruses to return to the wild bird AIV gene pool [24],[54], these emergent viruses must evolve as distinct eight-segment genome configurations within the new host. The ability of recent HP H5N1 AIV to cause spillover infections in wild birds is an unprecedented exception. Further, because humans represent a large and spatially mixed population, natural selection is able to act efficiently on individual subtypes [55]. Hence, a limited number of subtypes circulate within humans and evolve by antigenic drift to escape population immunity.

Notably, the recent Asian lineage HP H5N1 AIV strains are intermediate between these two contrasting influenza ecobiologies; a combination of large poultry populations allows natural selection to effectively drive rapid antigenic and genetic change within a single subtype [46],[56], while reassortment with the wild bird AIV gene pool facilitates the generation of new genome constellations [57]–[59]. Similar patterns have also been observed with the widely circulating H9N2 and H6N1 viruses in gallinaceous poultry in Eurasia [60],[61]. Previous analyses have also shown that recent HP H5N1 viruses had the highest evolutionary rates and selection pressures (d_N_/d_S_ ratios) as compared to other AIV lineages [46]. Consequently, these results underscore the importance of determining the mechanistic basis of how H5N1 has spread so successfully among a diverse range of both domestic and wild bird species.

## Materials and Methods

### Sample collection and virus isolation

The genomes of 167 influenza A virus isolates recovered from 14 species of wild Anseriformes located in four U.S. states (Alaska, Maryland, Missouri, Ohio) were sequenced for this study; viral isolates consisted of 29 hemagglutinin (HA) and neuraminidase (NA) combinations, including H1N1, H1N6, H1N9, H2N1, H3N1, H3N2, H3N6, H3N8, H4N2, H4N6, H4N8, H5N2, H6N1, H6N2, H6N5, H6N6, H6N8, H7N3, H7N8, H8N4, H10N7, H10N8, H11N1, H11N2, H11N3, H11N6, H11N8, H11N9, H12N5. Cloacal swabs were collected as previously described [39] from 1986–2005 as part of The Ohio State University's ongoing influenza A virus surveillance activities and in collaboration with many researchers in other states since 2001. A table listing the details of each isolate are available from the Influenza Virus Resource page (http://www.ncbi.nlm.nih.gov/genomes/FLU/Database/shipment.cgi). Avian influenza viruses were originally isolated using standard viral isolation procedures after 1–2 passages in 10-day-old embryonated chicken eggs (ECEs) [62]. Type A influenza virus was confirmed using commercially available diagnostic assays (Directigen Flu A Assay, Becton Dickinson Microbiology Systems, Cockeysville, MD) and isolates were subtyped at the National Veterinary Services Laboratories (NVSL), Animal and Plant Health Inspection Service, United States Department of Agriculture, Ames, Iowa, using standard hemagglutinin inhibition and neuraminidase inhibition testing procedures [51].

### Sequential Limiting Dilutions

Isolates for this investigation were generally selected from the viral archives based on antigenic diversity, clustering of recoveries, no evidence of antigenically mixed subtypes, and distribution over time. First- or second-egg-passage isolates in chorioallantoic fluid (CAF) were rapidly thawed from −80°C to room temperature, vortexed for 30 seconds and centrifuged at 1500 rpm for 10 minutes. Approximately 0.5 ml of CAF was drawn from the vial using a 26-gauge needle and subsequently passed through a 25 mm, 0.2 µm filter. Following filtration, a 10^−1^ CAF stock dilution was obtained by adding 0.2 ml filtered CAF to 1.8 ml Brain Heart Infusion Broth containing penicillin and streptomycin and vortexed for 30 seconds. Serial dilutions (10^−6^ maximum) were performed and 0.1 ml of each dilution was inoculated into each of four 10-day-old ECEs. After approximately 48 hours of incubation at 35°C/60% humidity, the inoculated eggs were chilled overnight and CAF was harvested from each egg and tested for hemagglutinating activity. The CAF from the last dilution positive for hemagglutinating activity was tested for the presence of type A influenza virus using the Directigen Flu A or Synbiotics Flu Detect Antigen Capture Test Strips™ (Synbiotics Corp., San Diego, CA). Hemagglutination titer assays were performed and CAF aliquots from the most dilute influenza A positive samples were stored at −80°C. If no endpoint titer was determined, the 10^−6^ CAF dilution was stored at −80°C and the procedure repeated utilizing 10^−4^ to 10^−9^ sequential dilutions.

### Preliminary molecular testing

Viral RNA was isolated from allantoic fluid using Trizol® Reagent (Invitrogen Corp., Carlsbad, CA) and transcribed into 20 µl of cDNA for a subset of samples [63]. Segment-specific universal primers designed to amplify partial and/or full-segments were initially used in RT-PCR assays to assess vRNA quality and RT-PCR primer specificity and sensitivity. Additionally, M13 sequencing tags (F primer: GTAAAACGACGGCCAG; R primer: CAGGAAACAGCTATGAC) were added to each primer set for ease of sequencing RT-PCR products in both forward and reverse directions.

### Primer design

For initiation of a high-throughput sequencing pipeline, a universal strategy for primer design was employed to ensure detection of multiple viral infections within a single sample. Primers were designed to semi-conserved areas of the six internal segments. For the segments encoding the external proteins, primers were designed from alignments of subsets of the 16 HA and 9 NA avian subtypes. Alignments were generated with MUSCLE [64] and visualized with BioEdit [65]. An M13 sequence tag was added to the 5′ end of each primer to be used for sequencing. Four sequencing reactions per run were analyzed on an agarose gel for quality control purposes. The sequence success rate of each primer pair was analyzed relative to the HA and NA subtype. Primers that did not perform well were altered or replaced. All primers and RT-PCR assay cycling conditions are available upon request.

### cDNA Synthesis and Sequencing

Influenza A virus isolates were amplified with the OneStep RT-PCR kit (Qiagen, Inc., Valencia, CA). Amplicons were sequenced in both the forward and reverse directions. Each amplicon was sequenced from each end using M13 primers (F primer: TGTAAAACGACGGCCAGT; R primer: CAGGAAACAGCTATGACC). Sequencing reactions were performed using Big Dye Terminator chemistry (Applied Biosystems, Foster City, CA) with 2 µl of template cDNA. Additional RT-PCR and sequencing was performed to close gaps and to increase coverage in low coverage or ambiguous regions. Sequencing reactions were analyzed on a 3730 ABI sequencer and sequences were assembled in a software pipeline developed specifically for this project.

### Sequence trimming and assembly

Once genomic sequence was obtained for an individual sample, reads for each segment were downloaded, trimmed to remove amplicon primer-linker sequence and low quality sequence, and assembled. A small genome assembly suite called Elvira (http://elvira.sourceforge.net/), based on the open-source Minimus assembler, was developed to automate these tasks. The Elvira software delivers exceptions including failed reads, failed amplicons, and insufficient coverage to a reference sequence (as obtained from GenBank), ambiguous consensus sequence calls, and low coverage areas. The avian influenza A sequences (with GenBank Accession numbers) produced from this ongoing study are available at http://www.ncbi.nlm.nih.gov/genomes/FLU/Database/shipment.cgi. The first 167 avian influenza genomes from this collection were submitted to GenBank and included in this study.

### Evolutionary analysis

The genomes of avian influenza virus newly determined here were combined with those already available on GenBank, particularly from recent large-scale surveys of viral biodiversity [38]. Sequences from viruses isolated before 1970, which may have been subjected to extensive laboratory passage, were excluded as were the large numbers of H5N1 sequences collected in recent years (a sample of H5N1 genomes, 1997–2005, were included for analysis). In total, 452 HA sequences and 473 NA sequences were used in analyses. For the internal protein-encoding segments (PB2, PB1, PA, NP, M, NS), a total of 407 genomes were analyzed (by considering a common data set we were able to investigate patterns of segment linkage, see below). For each data set, sequence alignments of the coding regions were created using MUSCLE [64] and adjusted manually using Se-Al [66] according to their amino acid sequence. In the case of HA and NA, some regions of the inter-subtype sequence alignment were extremely divergent such that they could not be aligned with certainty (HA signal peptide and cleavage site insertions in HPAI H5 or H7, and variable small stalk deletions in NA). Because of their potential to generate phylogenetic error, these small regions of ambiguity were deleted. This resulted in the following sequence alignments used for evolutionary analysis: PB2 = 2277 nt; PB1 = 2271 nt; PA = 2148 nt; HA = 1683 nt; NP = 1494 nt; NA = 1257 nt; M = 979 nt; NS = 835 nt. All sequence alignments are available from the authors on request. Nucleotide and amino acid identity was calculated using Megalign (Lasergene 7.2, DNAStar, Madison, WI).

Using these alignments, maximum likelihood (ML) trees were inferred using PAUP* [67], based on the best-fit models of nucleotide substitution models determined by MODELTEST [68]. In most cases, the preferred model of nucleotide substitution was GTR+I+Γ_4_, or a close relative. For each of these trees, the reliability of all phylogenetic groupings was determined through a bootstrap resampling analysis (1000 pseudo-replicates of neighbor-joining trees estimated under the ML substitution model).

We employed a maximum likelihood method to assess the extent of phylogenetic congruence, indicative of reassortment [48]. To reduce any bias in phylogenetic structure caused by geographic segregation, only isolates from North American flyways were used in analyses of the internal gene segments. Briefly, ML trees for each internal gene segment were estimated as described above. Next, the log likelihood (-LnL) of each of the ML trees was estimated on each gene segment data set in turn, optimizing branch lengths under the ML substitution model in every case. The topological similarity between each gene segment tree on each data set was then determined by compared the difference in likelihood among them (Δ-LnL). Clearly, the greater the similarity in topology (congruence) among the trees for each segment, the closer their likelihood scores and so the more likely they are to be linked. To put the distribution of Δ-LnL values in context, we constructed 500 random trees for each data set and optimized their branch lengths in the same manner. If any of the Δ-LnL values among the ML trees falls within the random distribution then we can conclude that the gene segments in question are in complete linkage equilibrium. All these analyses were conducted using PAUP* package [67].

## Supporting Information

Figure S1 Maximum likelihood tree of the HA gene of 452 isolates of avian influenza A virus, including representatives of all 16 subtypes. Sequences are color-coded according to HA subtype (see Figure 1). Internal branches are color-coded to reflect the flyway from which the viruses were sampled; North American flyway in red, Eurasian flyway in blue. Bootstrap values above 70% are shown next to the relevant branches. Branch lengths are scaled according to the number of nucleotide substitutions per site.
(1.16 MB EPS)

Figure S2 Maximum likelihood tree of the NA gene of 473 isolates of avian influenza A virus, including representatives of all 9 subtypes. Sequences are color-coded according to HA subtype (see Figure 1), with the mix of colors highlighting the frequency of reassortment. Internal branches are color-coded to reflect the flyway from which the viruses were sampled; North American flyway in red, Eurasian flyway in blue. Bootstrap values above 70% are shown next to the relevant branches. Branch lengths are scaled according to the number of nucleotide substitutions per site.
(1.02 MB EPS)

Figure S3 Maximum likelihood tree of the PB2 gene of avian influenza A viruses. Sequences are color-coded according to HA subtype. Internal branches are color-coded to reflect the flyway from which the viruses were samples: North American flyway in red, Eurasian flyway in blue. Bootstrap values above 70% are shown next to relevant branches.
(0.84 MB EPS)

Figure S4 Maximum likelihood tree of the PB1 gene of avian influenza A viruses. Sequences are color-coded according to HA subtype. Internal branches are color-coded to reflect the flyway from which the viruses were samples: North American flyway in red, Eurasian flyway in blue. Bootstrap values above 70% are shown next to relevant branches.
(0.84 MB EPS)

Figure S5 Maximum likelihood tree of the PA gene of avian influenza A viruses. Sequences are color-coded according to HA subtype. Internal branches are color-coded to reflect the flyway from which the viruses were samples: North American flyway in red, Eurasian flyway in blue. Bootstrap values above 70% are shown next to relevant branches.
(0.83 MB EPS)

Figure S6 Maximum likelihood tree of the NP gene of avian influenza A viruses. Sequences are color-coded according to HA subtype. Internal branches are color-coded to reflect the flyway from which the viruses were samples: North American flyway in red, Eurasian flyway in blue. Bootstrap values above 70% are shown next to relevant branches.
(0.79 MB EPS)

Figure S7 Maximum likelihood tree of the M genes of avian influenza A viruses. Sequences are color-coded according to HA subtype. Internal branches are color-coded to reflect the flyway from which the viruses were samples: North American flyway in red, Eurasian flyway in blue. Bootstrap values above 70% are shown next to relevant branches.
(0.79 MB EPS)

Figure S8 Maximum likelihood tree of the NS genes of avian influenza A viruses. Sequences are color-coded according to HA subtype. Internal branches are color-coded to reflect the flyway from which the viruses were samples: North American flyway in red, Eurasian flyway in blue. Bootstrap values above 70% are shown next to relevant branches.
(0.83 MB EPS)

Table S1 Sequencing results for 167 complete genomes of 29 subtypes of avian influenza A viruses.
(0.06 MB DOC)
