# Supplementary material for: The Evolutionary Genetics and Emergence of Avian Influenza Viruses in Wild Birds
Source: PLoS Pathog. 2008 May 30;4(5):e1000076. doi: 10.1371/journal.ppat.1000076 (PMC2387073; doi:10.1371/journal.ppat.1000076)
Supplement: Table S1 — Sequencing results for 167 complete genomes of 29 subtypes of avian influenza A viruses. (0.06 MB DOC) [file ppat.1000076.s009.doc]

**The Evolutionary Genetics and Emergence of**

**Avian Influenza Viruses in Wild Birds**

Vivien G. Dugan, Rubing Chen, David J. Spiro, Naomi Sengamalay, Jennifer Zaborsky, Elodie Ghedin, Jacqueline Nolting, David E. Swayne, Jonathan A. Runstadler,

George M. Happ, Dennis A. Senne, Ruixue Wang, Richard D. Slemons,

Edward C. Holmes, Jeffery K. Taubenberger

**Supplementary Files**

**Table S1:** Sequencing results for 167 complete genomes of 29 subtypes of avian influenza A viruses.

| Segment | Length (nt) | Coding region (nt) | Total finished  sequence (nt) | Finished sequence per segment  (ave. nt) | Coding completeness (ave. %) | Average fold segment coverage |
| --- | --- | --- | --- | --- | --- | --- |
| PB2 | 2341 | 2277 | 384654 | 2303 | 100 | 7.8X |
| PB1 | 2341 | 2271 | 384116 | 2300 | 100 | 7.7X |
| PA | 2233 | 2148 | 367273 | 2199 | 100 | 6.9X |
| HA | 1728-1779 | 1695* | 287552 | 1712 | 100 | 11.9X |
| NP | 1565 | 1494 | 255451 | 1530 | 100 | 8.6X |
| NA | 1398-1469 | 1410* | 239623 | 1410 | 100 | 7.9X |
| M | 1027 | 982 | 164882 | 987 | 100 | 7.6X |
| NS | 890 | 838 | 142534 | 854 | 100 | 7.6X |
| Total | 13523-13645 | 13115 | 2226085 | 13295 | 100 | 8.3X |

*Average in this study
